# Primary extragonadal germ cell tumours: Real‐world data from a high‐volume centre

**DOI:** 10.1002/bco2.70202

**Published:** 2026-04-16

**Authors:** Marc Kidess, Marcus Hentrich, Franz Aschl, Oksana Chernova, Sebastian Beckstein, Peter Bojko, Sebastian Schulz, Julia Neitz, Julian Hermans, Yannic Volz, Benedikt Ebner, Patrick Keller, Maria Apfelbeck, Julian Marcon, Philipp Weinhold, Christian G. Stief, Lennert Eismann, Michael Chaloupka

**Affiliations:** ^1^ Department of Urology LMU University Hospital of Munich Munich Germany; ^2^ Department of Medicine III Red Cross Hospital Munich Munich Germany; ^3^ School of Computation, Information and Technology Technical University of Munich Munich Germany

**Keywords:** extragonadal, germ cell tumour, mediastinal, non‐seminoma, seminoma cancer

## Abstract

**Objectives:**

To provide improved evidence for treatment recommendations, this study analysed real‐world data on the characteristics, treatment and prognosis of primary extragonadal germ cell tumours (EGCTs), a very rare cancer entity that shares histological features with testicular GCTs (TGCT).

**Materials and methods:**

This retrospective study analysed data from 34 patients with mediastinal or retroperitoneal EGCTs treated at a high‐volume centre between 2015 and 2025. Probability of overall survival (OS) and relapse‐free survival (RFS) was assessed using Kaplan–Meier curves.

**Results:**

A primary retroperitoneal and primary mediastinal GCT was diagnosed in 23 (68%) and 11 (32%) patients, respectively. Median patient age was 41.1 years, and 62% were non‐seminomatous in origin. Thirty‐three (97%) patients received chemotherapy according to the IGCCCG prognostic group, and 15 (44%) underwent surgical intervention (4 primary RPLND, 8 post‐chemotherapy RPLND, 3 secondary orchiectomy). Thirteen patients (44.9%) who completed chemotherapy experienced a relapse after a median of 0.79 years, and six of them died. In patients with primary mediastinal seminoma, 3‐year RFS and OS were 100% as compared to 71% 3‐year OS in patients with primary retroperitoneal non‐seminoma and 46% 3‐year RFS in patients with primary mediastinal non‐seminoma.

**Conclusion:**

The prognosis for primary mediastinal seminoma appears favourable, while non‐seminomatous EGCT shows poorer outcomes. Additionally, primary retroperitoneal seminomas demonstrate promising survival rates, whereas primary retroperitoneal non‐seminomas demonstrate the lowest OS rates.

## 1 INTRODUCTION

Germ cell tumours (GCTs) are mostly (95%) located in the testicles (testicular germ cell tumours [TGCT]), making them the most common neoplasm among young men aged 15–40 years.^1^ The prognosis is very good with a 5‐year survival rate of 95%.^2^ GCTs can be classified as seminomas or non‐seminomas, the latter comprising teratomas, embryonal carcinomas, choriocarcinomas and yolk sac tumours. In a subgroup of about 5% of patients, the origin of GCTs is extragonadal (EGCTs), referring to neoplasms that display histologies associated with gonadal origin^3^: According to a previous study, 54% of EGCTs are primary mediastinal, and 45% primary retroperitoneal with other extragonadal sites being exceedingly rare.^4^

Although EGCT are similar to testicular GCT (TGCT) in terms of histology, serology and cytogenetics, the proportion of patients with non‐seminomas was reported to be 83% in an international collaborative study,^4^ which is much higher than the approximately 44% proportion of non‐seminomas in TGCT.^5^ Differences in clinical behaviour may also be based on biological differences^6^ as studies have suggested that EGCT might result from a malignant transformation of germ cells that were either maldistributed during embryonic development or from germ cells that naturally occur at extragonadal locations to control immunological processes or other organ functions.^3^ Notably, primary mediastinal non‐seminomas have a particular unfavourable prognosis.^6^, ^7^, ^8^ In clinical practice, it might be difficult to distinguish between extragonadal metastasis from an already regressed primary testis lesion (‘burn‐out’ primary) and a real EGCT.^3^, ^9^ Especially, retroperitoneal EGCTs may be associated with a regressed primary testicular lesion.^10^

Depending on the localization of the EGCT, patients may present with symptoms like dyspnoea, chest pain, fever, weight loss, abdominal or back pain.^4^, ^5^ Only limited data are available on studies specifically addressing EGCT management. The German testicular cancer guideline addresses EGCT management as follows^11^: Diagnostics include tumour markers for testicular cancer (alpha‐fetoprotein, lactate dehydrogenase and human chorionic gonadotropin), sonography and computed tomography as well as biopsy of the tumour masses. Classification is performed according to the IGCCCG classification system.^12^ Therapeutic strategies are similar to those for metastatic testicular cancer. If a regressed primary testicular lesion is suspected, orchiectomy can be performed.

This study aims to provide insight into real‐world data on characteristics, therapy and prognosis of EGCT treated in two experienced institutions.

## 2 MATERIALS AND METHODS

### 2.1 Patient population

A retrospective analysis was performed on GCT patients who received therapy at Red Cross Hospital and/or LMU University Hospital between January 2015 and May 2025. Both institutions are reference centres for germ cell cancer. The dataset contained information on 429 patients with gonadal and extragonadal GCTs and included data on demographics, histopathology, stage and management. For this study, we extracted data on patients presenting with mediastinal or retroperitoneal EGCT. This study was approved by the LMU ethics board (reference 25‐0341) and performed in compliance with the principles of the Declaration of Helsinki and its subsequent amendments.

### 2.2 Follow‐up

If routine follow‐up was not performed at the treating institutions, a follow‐up assessment was conducted via telephone interviews with referring physicians and/or patients in August 2025. Items included the date of the last clinical follow‐up, date and cause of death, latest imaging and tumour marker results, persistence of treatment side effects and occurrence of new comorbidities or tumour marker relapse.

### 2.3 Statistical analysis

Statistical analysis was conducted in accordance with the *Guidelines for Reporting of Statistics for Clinical Research in Urology*.^13^ Data processing was performed using R studios. Descriptive data are presented as the median with the 25th percentile and 75th percentile ([Q1; Q3]) for continuous variables and as absolute numbers with the corresponding percentages for categorical variables. For the follow‐up analysis, we examined the overall survival (OS) and relapse‐free survival (RFS) probabilities. OS was defined as the time from the start of follow‐up (the last day of the last course of chemotherapy) to death from any cause. RFS was defined as the time from the beginning of the follow‐up period to disease recurrence or death from any cause. Survival probabilities were graphically demonstrated using Kaplan–Meier plots. Due to the inherent limitations of small subgroup cohorts and wide 95% confidence intervals, we restricted our primary outcomes to 1‐year and 3‐year survival estimates. This approach avoids unreliable long‐term projections beyond the median follow‐up period of 1–2 years. Due to the small sample size, statistical testing for significance was not performed. Furthermore, also due to the small sample size, complete response/remission (CR) and no evidence of disease (NED) were analysed as equivalent.

## 3 RESULTS

### 3.1 Baseline characteristics

Thirty‐four out of 429 patients (7.9%) were diagnosed with EGCT and included in the present analysis. Baseline characteristics and preconditions are displayed in Table 1.

**TABLE 1:** Baseline characteristics.

*N* = 34 (100%)	
Age (median [Q1; Q3])	41.1 years [30.0; 55.0]
Primary tumour site	
Retroperitoneal (*n*; %)	23 (67.6%)
Seminoma	10 (43.5%)
Non‐seminoma	13 (56.5%)
Mediastinal (*n*; %)	11 (32.4%)
Seminoma	3 (27.3%)
Non‐seminoma	8 (72.7%)
Pathology	
Seminoma (*n*; %)	13 (38.2%)
Non‐seminoma (*n*; %)	21 (61.7%)
Choriocarcinoma (*n*; %)	1 (4.8%)
Yolk sack tumour (*n*; %)	2 (9.5%)
Embryonal carcinoma (*n*; %)	10 (47.6%)
Teratoma (*n*; %)	7 (33.3%)
N/A (*n*; %)	1 (4.8%)
Clinical stage	
II A (*n*; %)	0 (0%)
II B (*n*; %)	1 (2.9%)
II C (*n*; %)	8 (23.5%)
III A (*n*; %)	5 (14.7%)
III B (*n*; %)	5 (14.7%)
III C (*n*; %)	11 (32.4%)
N/A (*n*; %)	4 (11.8%)
IGCCCG prognostic group	
Good prognosis (*n*; %)	15 (44.1%)
Intermediate prognosis (*n*; %)	5 (14.7%)
Poor prognosis (*n*; %)	13 (38.2%)
N/A (*n*; %)	1 (2.9%)
Tumour marker at diagnosis	
AFP (median [Q1; Q3])	5.3 μg/L [2.81; 870.0]
HCG (median [Q1; Q3])	33.5 IU/L [2.00; 440.0]
LDH (median [Q1; Q3])	419 U/L [331.0; 1010.0]
Preconditions	
Smoking (*n*; %)	4 (11.8%)
Hypertension (*n*; %)	5 (14.7%)
Diabetes mellitus type 2 (*n*; %)	2 (5.9%)
Hepatitis or HIV (*n*; %)	0 (0%)
Others (*n*; %)	27 (79.4%)

Abbreviations: AFP, alpha‐fetoprotein; HCG, human chorionic gonadotropin; IGCCCG, International Germ Cell Cancer Consensus Classification; LDH, lactate dehydrogenase; N/A, no answer.

The median age was 41.1 years. Twenty‐three patients (67.6%) had a primary retroperitoneal GCT, of which 13 (56.5%) were non‐seminomas. Eight of 11 patients (72.7%) with primary mediastinal GCT were non‐seminomatous in origin. Among non‐seminomas, the most common pathology was embryonal carcinoma (*n* = 10, 47.6%), followed by teratoma (*n* = 7, 33.3%). Stage II and stage III disease were diagnosed in nine (26.5%) and 21 (61.8%) patients, respectively. Fifteen (44.1%), 4 (15.7%) and 13 (38.2%) patients had good, intermediate and poor prognosis according to the IGCCCG classification. Most patients were diagnosed by needle biopsy (*n* = 26; 76.5%), four (11.8%) by laparoscopic extirpation of suspicious lymph nodes, and one patient underwent open tumour dissection. In one patient with far advanced disease, the diagnosis was made based on clinical presentation combined with elevated tumour markers. Four (11.8%) patients had smoked at some time point in their lives. Five patients (14.7%) suffered from hypertension.

### 3.2 Treatment

Table 2 displays initial treatment modalities.

**TABLE 2:** Treatment modalities.

Treatment (*N* = 34)	
Chemotherapy (*n*; %)	33 (97.1%)
3 cycles PEB (*n*; %)	6 (18.2%)
Good prognosis	6 (100%)
4 cycles PEB (*n*; %)	13 (39.4%)
Good prognosis	1 (7.7%)
Intermediate prognosis	3 (23.1%)
Poor prognosis	8 (61.5%)
N/A	1 (7.7%)
3 cycles PEI (*n*; %)	2 (6.1%)
Good prognosis	1 (50%)
Poor prognosis	1 (50%)
4 cycles PEI (*n*; %)	4 (12.1%)
Intermediate prognosis	2 (50%)
Poor prognosis	2 (50%)
4 cycles PE (n; %)	6 (18.2%)
Good prognosis	5 (83.3%)
Poor prognosis	1 (1.7%)
Others	1 (3.0%)
N/A (*n*; %)	1 (3.0%)
Surgical intervention (*n*; %)	15 (44.2%)
Secondary orchiectomy (*n*; %)	3 (20.0%)
Primary RPLND (*n*; %)	4 (26.7%)
Seminoma	2 (50%)
Non‐seminoma	2 (50%)
Post‐chemotherapy RPLND (*n*; %)	8 (53.3%)
Seminoma	1 (12.5%)
Non‐seminoma	4 (50%)
No malignancy/necrosis	3 (37.5%)
Other (*n*; %)	1 (6.7%)

Abbreviations: N/A, no answer; PE, cisplatin + etoposide; PEB, cisplatin + etoposide + bleomycin; PEI, cisplatin + etoposide + ifosfamide.

A total of 33 patients (97.1%) received chemotherapy, which was performed after primary retroperitoneal lymph node dissection (RPLND) in four cases. One patient solely underwent primary RPLND. Among patients undergoing chemotherapy, 13 (39.4%) received four cycles of PEB, and six patients each (18.2%) underwent four cycles of PE or three cycles of PEB. Table 2 shows chemotherapy strategies in the context of IGCCCG prognosis groups. Fifteen patients (44.2%) underwent some type of surgical intervention, of which four (26.7%) underwent primary RPLND and eight (53.3%) post‐chemotherapy RPLND. In five (62.5%) of the latter group, vital tumour tissue was found in the specimen. A secondary testicular biopsy was performed on three patients, but no malignancy was detected in any specimens. Three patients underwent secondary orchiectomy. One patient (33.3%) had seminoma in the testicular specimen, but it remained unclear whether this was a de novo seminoma.

### 3.3 Survival

Table 3 displays the baseline characteristics of the patients included in the follow‐up. Median follow‐up for RFS was 1.3 (0.4; 7.2) years; median follow‐up for OS was 2.1 (0.7; 8.1) years. Five patients were excluded due to a lack of follow‐up data. Thirteen patients (44.9%) who completed chemotherapy experienced a relapse after a median time of 0.79 years (0.16; 8.14), and six of them (46.2%) died of relapsed/refractory disease. One patient died during the first course of chemotherapy due to neutropenic sepsis.

**TABLE 3:** Baseline characteristics of the patients included in the follow‐up.

*N* = 29 (100%)	
Age at start of follow up median [Q1; Q3]	36 [30; 56] years
Follow‐up time among survivors median [Q1; Q3]	3.05 [0.57; 7.65] years
Primary tumour site	
Retroperitoneal (*n*; %)	19 (65.5%)
Seminoma	8 (42.1%)
Non‐seminoma	11 (57.9%)
Mediastinal (*n*; %)	10 (34.5%)
Seminoma	3 (30%)
Non‐seminoma	7 (70%)
Pathology	
Seminoma (*n*; %)	11 (37.9%)
Non‐seminoma (*n*; %)	18 (62.1%)
Clinical stage	
II (*n*; %)	7 (24.1%)
II A (*n*; %)	0 (0%)
II B (*n*; %)	1 (3.4%)
II C (*n*; %)	6 (20.7%)
III (*n*; %)	20 (68.9%)
III A (n; %)	4 (13.8%)
III B (n; %)	5 (17.2%)
III C (n; %)	11 (37.9%)
N/A (*n*; %)	2 (6.9%)
IGCCCG prognosis group	
Good prognosis (*n*; %)	12 (41.4%)
Intermediate prognosis (*n*; %)	4 (13.8%)
Poor prognosis (*n*; %)	12 (41.4%)
N/A (*n*; %)	1 (3.4%)

### 3.4 Survival by subgroups

**FIGURE 1:**
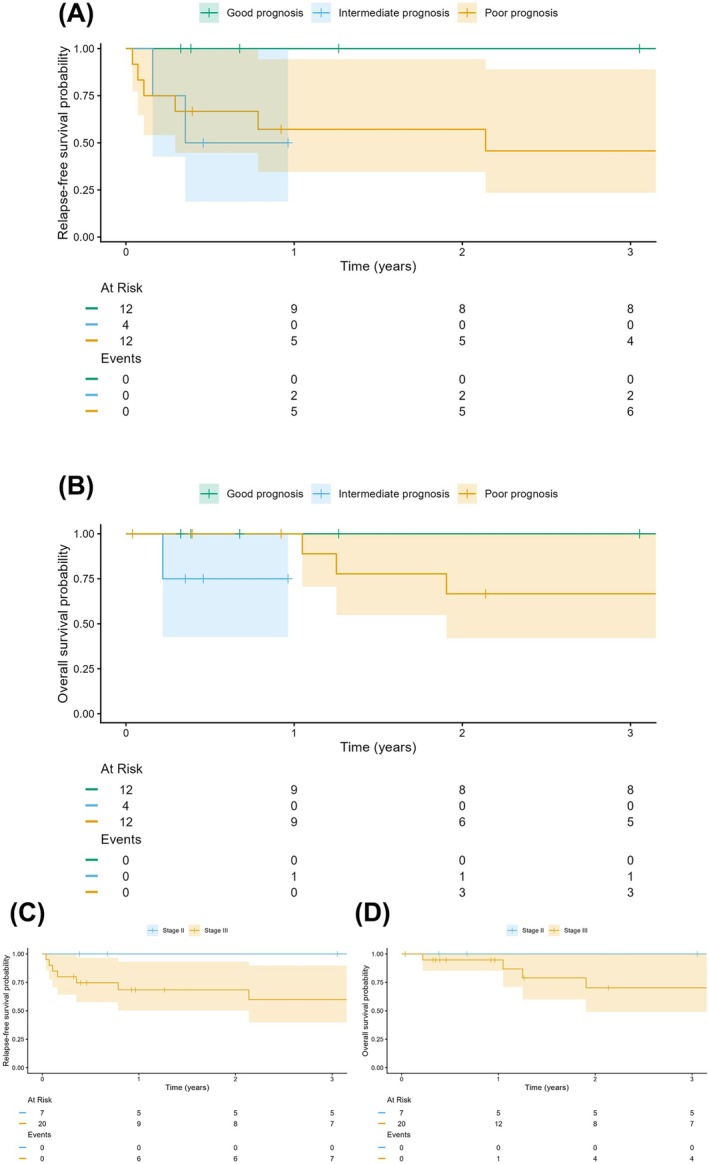
Kaplan–Meier curves comparing patients in IGCCCG good (green), intermediate (blue) and poor prognosis (yellow) group in terms of relapse‐free survival (RFS) (A) and overall survival (OS) (B). The comparison of RFS and OS for stages II (blue) and III (yellow) is displayed in (C) and (D), respectively.

RFS varied by IGCCCG prognosis group. The 1‐year RFS was 100% for the good prognosis group, 50% (95% CI: 19%–100%) for the intermediate group and 57% (95% CI: 35%–94%) for the poor prognosis group. At 3 years, RFS remained 100% for the good prognosis group, while the poor prognosis group declined to 46% (95% CI: 23%–89%). Comparisons involving the intermediate group are limited by the small sample size and a lack of follow‐up beyond the first year, as indicated by the ‘At Risk’ table (Figure 1A). One‐year OS was 100% for both the good (*n* = 12) and poor (*n* = 12) prognosis groups. In contrast, the intermediate prognosis group (*n* = 4) demonstrated a 75% OS (95% CI: 43%–100%) following a single early mortality event. By year 3, OS remained 100% for the good prognosis group but declined to 67% (95% CI: 42%–100%) for the poor prognosis group. Survival estimates for the intermediate prognosis group beyond 1 year could not be reliably determined due to the absence of patients at risk after the 12‐month interval (Figure 1B).

The 3‐year RFS for stage II patients (*n* = 7) was 100%, with no relapse events recorded during the follow‐up period. In contrast, stage III patients (*n* = 20) demonstrated a 1‐year RFS of 68% (95% CI: 50%–93%) and a 3‐year RFS of 60% (95% CI: 40%–90%). The majority of relapse events in the stage III cohort occurred within the first year of follow‐up (Figure 1C). OS was high at 1 year across both stages (stage II: 100%; stage III: 95%). However, long‐term outcomes diverged by year 3, with stage II maintaining 100% OS compared to 70% (95% CI: 49%–100%) for stage III. No mortality was observed in the stage II group (*n* = 7), whereas four mortality events occurred in the stage III group (*n* = 20) during the 3‐year follow‐up period (Figure 1D).

**FIGURE 2:**
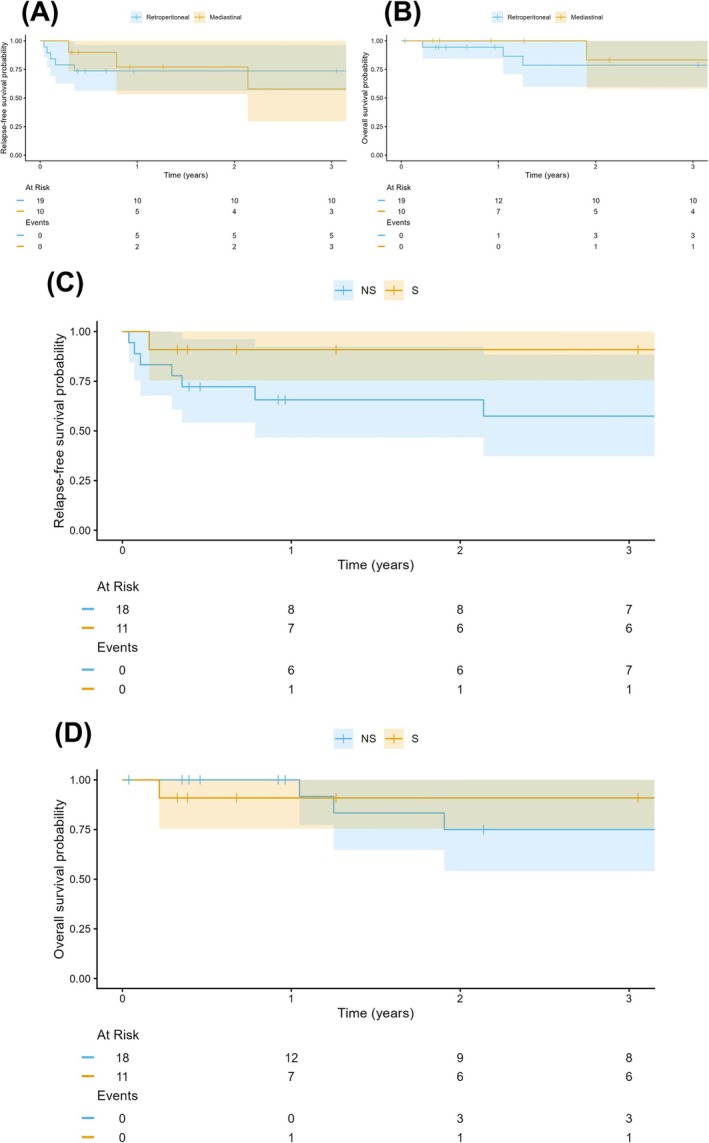
Kaplan–Meier curves comparing relapse‐free survival (A) and overall survival (B) of patients with primary retroperitoneal GCT (blue) and mediastinal GCT (yellow) as well as relapse‐free survival (C) and overall survival (D) of seminoma (S; yellow) and non‐seminoma (NS, blue) patients.

RFS was influenced by pathology. Seminoma patients (*n* = 11) demonstrated excellent outcomes with a 1‐year and 3‐year RFS of 91% (95% CI: 75%–100%), with only a single relapse event recorded early in the follow‐up period. In contrast, the non‐seminoma group (*n* = 18) experienced a higher rate of early recurrence, with a 1‐year RFS of 66% (95% CI: 47%–92%), which further declined to 57% (95% CI: 37%–88%) by year 3. The majority of relapses in the non‐seminoma cohort occurred within the first 12 months (Figure 2C). At the 1‐year mark, OS was 100% for the non‐seminoma patients (*n* = 18) and 91% (95% CI: 75%–100%) for the seminoma cohort (*n* = 11). However, late mortality events were observed in the non‐seminoma group, with OS declining to 75% (95% CI: 54%–100%) by year 3. In contrast, the seminoma group remained stable at 91% survival through the 36‐month study endpoint (Figure 2D).

Tumour location appeared to influence the temporal pattern of RFS. In the retroperitoneal cohort (*n* = 19), RFS was 74% (95% CI: 56%–96%) at 1 year and remained stable through the 3‐year mark, with all relapse events occurring within the first 12 months of follow‐up. Conversely, the mediastinal patients (*n* = 10) demonstrated a 1‐year RFS of 77% (95% CI: 53%–100%) but experienced late recurrence, leading to a decline in RFS to 58% (95% CI: 29%–100%) by year 3. While initial outcomes were comparable, the divergence at the 36‐month interval suggests a potentially higher risk of late relapse for patients with mediastinal primary tumours (Figure 2A). OS was high across both primary tumour locations at the 1‐year mark, with 94% (95% CI: 84%–100%) survival in the retroperitoneal group (*n* = 19) and 100% survival in the mediastinal group (*n* = 10). At 3 years, OS remained comparable between the two cohorts, at 79% (95% CI: 60%–100%) for retroperitoneal GCT and 83% (95% CI: 58%–100%) for mediastinal GCT. While the mediastinal group maintained a higher survival probability throughout the follow‐up period, this was based on a single mortality event compared to three events in the retroperitoneal group (Figure 2B).

**FIGURE 3:**
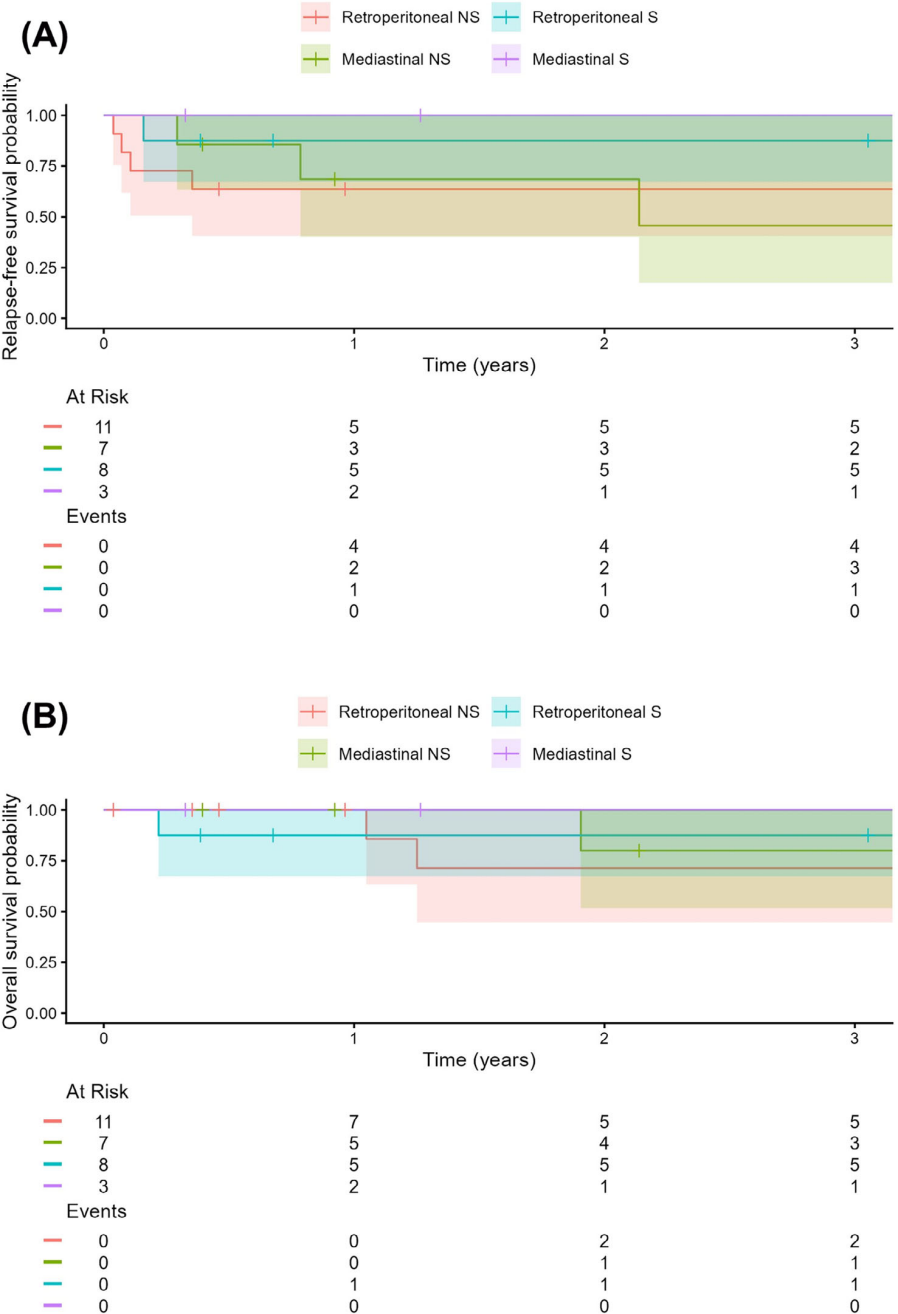
Kaplan–Meier curves comparing relapse‐free survival (A) and overall survival (B) of patients with primary retroperitoneal non‐seminoma (NS; red) and seminoma (S; blue) and primary mediastinal NS (green) and S (purple).

Stratification by pathology and primary tumour location revealed distinct relapse patterns. Within the non‐seminoma (NS) cohort, patients with mediastinal GCT (*n* = 7) demonstrated the highest risk for late recurrence, with RFS dropping from 69% (95% CI: 40%–100%) at year 1 to 46% (95% CI: 17%–100%) by year 3. Conversely, the NS retroperitoneal group (*n* = 11) experienced all relapse events within the first year, maintaining a stable RFS of 64% (95% CI: 41%–99%) through the 3‐year mark. Patients with seminoma (S) showed superior stability across both locations. The 3‐year RFS was 100% for the mediastinal subgroup (*n* = 3) and 88% (95% CI: 67%–100%) for the retroperitoneal subgroup (*n* = 8) (Figure 3A).

At the 1‐year mark, OS was high across all subgroups, reaching 100% for both non‐seminomatous subgroups and the subgroup with mediastinal seminomatous GCT, while the patients with retroperitoneal seminomatous GCT demonstrated an OS of 88% (95% CI: 67%–100%). However, a divergence in outcomes was observed by year 3. The subgroup with retroperitoneal NS (*n* = 11) experienced the most significant decline, with OS dropping to 71% (95% CI: 45%–100%). Patients with mediastinal NS (*n* = 7) showed slightly higher resilience with a 3‐year OS of 80% (95% CI: 52%–100%). In contrast, survival remained stable in the seminoma cohorts; the group with retroperitoneal seminomatous GCT maintained 88% OS, and the patients with mediastinal seminoma (*n* = 3) recorded no mortality events, maintaining 100% OS through the 3‐year endpoint (Figure 3B).

## 4 DISCUSSION

EGCT is a rare form of germinal cancer, accounting for 5% of GCTs.^3^ This reflects the lack of specific recommendations from essential international guidelines such as the European Association of Urology (EAU), the American Urological Association (AUA) or the European Society for Medical Oncology (ESMO). Further, it may be difficult in daily practice to distinguish between extragonadal metastasis from a regressed primary testicular lesion and a true EGCT, not least because of differences in clinical behaviour that suggest different biology.^3^, ^6^, ^9^ This study therefore focuses on the epidemiology and on survival rates of patients with EGCT. We found that the majority of EGCTs were located in the retroperitoneum and were predominantly non‐seminomatous. Patients with a poor prognosis had the highest relapse rate, and relapse increased the risk of death. Patients with seminoma fared better than those with non‐seminoma, and the worst survival outcome was detected in patients suffering from primary retroperitoneal non‐seminoma.

Of note, the median patient age was 41 years, which is markedly higher than the median age of 30 years as reported in a study on 635 patients with EGCT published in 2002.^4^ This discrepancy may be explained by the increasing age at initial diagnosis among TC patients as observed over the last few decades. Yamashita et al. demonstrated that the median age at diagnosis increased continuously from 28 to 38 years between 1980 and 2019.^14^ Our observation that retroperitoneal EGCTs are more common than mediastinal EGCTs is inconsistent with data from previous studies showing primary mediastinal GCTs to be more common than primary retroperitoneal GCTs.^4^, ^15^ However, this difference may be due to the small sample size of the present study. Regarding histopathology, our data are in line with findings from the Surveillance, Epidemiology, and End Results‐based (SEER) programme that identified non‐seminomatous EGCT as the predominant pathology (56%), which is similar to the 60% share of non‐seminomas in our study.^15^ In our study, 69% and 41% of patients were diagnosed with stage III disease and belonged to the poor prognosis group, respectively. While the latter is largely due to the unfavourable prognosis of primary mediastinal non‐seminoma, the high proportion of patients diagnosed with stage III disease may be due to non‐specific symptoms depending on tumour localization, which may lead to delayed diagnosis.^4^

It is not surprising that the vast majority of patients (97%) received chemotherapy. Treatment strategies for EGCT are similar to those for metastatic testicular cancer largely depending on the IGCCG prognostic group.^11^ Patients with good prognosis should receive three courses of PEB or four courses of EP, while those with intermediate and poor prognosis should be treated with receive four courses of PEB or PEI.^11^, ^16^ For patients with stage IIA/B seminoma, radiotherapy may also be the treatment of choice. The slight deviations from the therapy recommendations that were made in some patients in our cohort were mainly due to individual patient characteristics. For example, one patient each received four cycles of PE or only three cycles of PEI despite belonging to the poor prognostic group as both patients were not able to tolerate a higher chemotherapy intensity.

It is important to note that in patients with primary extragonadal seminoma, chemotherapy is the preferred option to radiotherapy. Results from a previous study showed that patients with seminomatous EGCT who received either cisplatin‐based chemotherapy alone or chemotherapy plus radiotherapy had better progression‐free survival rates than those who received primary radiotherapy only.^17^

Different to other studies,^6^, ^8^ we observed the worst OS in patients with retroperitoneal NS but observed the worst RFS in mediastinal NS. These results suggest that while early survival is excellent regardless of subtype, non‐seminoma pathology—particularly in retroperitoneal primaries—may be associated with a higher risk of late‐term mortality. As with other stratified analyses, these findings are limited by small subgroup sizes and wide confidence intervals. Whether or not primary high‐dose chemotherapy (HD‐CT) should be applied or not is a matter of debate. Encouraging results were reported from a prospective multicentre trial on 28 patients with primary mediastinal NS undergoing sequential HD‐CT followed by autologous stem cell transplants (ASCT).^18^ Nineteen patients (68%) obtained a disease‐free status with a PFS and OS of 64% and 68%, respectively.^18^ In a recent study by the European Bone Marrow Transplantation (EBMT) that included 24 patients receiving upfront HD‐CT with ASCT for primary non‐seminoma mediastinal GCT, the 5‐year‐PFS was reported to be 51.8%, and the 5‐year OS to be 51.3%.^19^ However, HD‐CT plus ASCT was not superior to conventional dose CT in three prospective randomized trials on unselected patients with poor prognosis GCT.^20^, ^21^, ^22^

In the present study, 15 of 34 patients (44%) underwent some type of surgical intervention with post‐chemotherapy RPLND performed in 8 of 15 patients (53%). In contrast, only 25%–30% of patients with non‐seminomatous TC undergo residual tumour dissection after chemotherapy.^23^ Post‐chemotherapy RPLND is recommended for non‐seminoma patients with residual tumour masses larger than 1 cm, while primary RPLND is recommended for patients with pure teratoma.^16^ Nevertheless, as emphasized by our study, surgery is a critical part of the treatment strategy for EGCT, especially for removing residual disease after CT. It is important to note that only three patients underwent secondary orchiectomy. Some studies suggest that EGCTs are metastases of ‘burned‐out’ testicular tumours. Fossa et al. demonstrated that carcinoma in situ (CIS) was present in 31% of patients with EGCTs, particularly in those with retroperitoneal tumours.^24^ The authors concluded that bilateral testicular biopsies should be performed in patients with EGCTs. However, in our study, a secondary testicular biopsy was performed on three patients without detection of malignancy.

It is not unexpected that RFS and OS were markedly better in patients with seminomatous EGCT compared to non‐seminomatous EGCT (3‐year RFS: 91% vs. 57%; 3‐year OS: 91% vs. 75%) with mediastinal seminoma demonstrating 100% 3‐year RFS and OS. Long‐term outcomes in patients with primary EGCT largely depend on histopathology and the primary site. In a large retrospective international study on EGCT, patients with retroperitoneal non‐seminomatous EGCT had better survival rates than those with mediastinal non‐seminomatous EGCT (63% vs. 49%, *p* < 0.0006).^4^ By contrast, no differences in overall survival rates (88%) were observed in patients with seminomatous EGCT regardless of the primary tumour location.^4^ Another study by Makino et al. showed that the 5‐year overall survival rate for patients with seminomatous and non‐seminomatous EGCT was 100% and 44%, respectively (*p* = 0.29) with 5‐year overall survival rates of 100%, 40% and 40% in the good, intermediate and poor prognosis groups, respectively (*p* = 0.18).^25^ In contrast, our findings suggest that while seminomatous EGCT exhibit consistent favourable outcomes regardless of location, non‐seminomatous mediastinal EGCT may require more intensive long‐term surveillance due to the risk of delayed relapse.

This study is not devoid of limitations. The small number of patients does not allow for meaningful testing for significance. Studies with larger patient numbers are necessary to strengthen the results. Our observations are limited by the small subgroup size and should be interpreted as preliminary, which is caused by the rareness of this cancer entity. Further, the retrospective study design negatively impacts the study's power. Finally, as the median follow‐up time is only 1.3–2.1 years, longer follow‐up will be necessary to strengthen the significance of our results.

## 5 CONCLUSION

In this study on EGCT, relapse‐free survival and overall survival largely depend on primary tumour localization, histopathology and the IGCCCG prognostic group. Patients with non‐seminomatous EGCT had the worst 3‐year RFS and OS rates, and relapses increase the risk of death. Further research with larger patient numbers is necessary to improve guideline recommendations for this rare cancer.

## AUTHOR CONTRIBUTIONS

**Marc Kidess:** conception and design; acquisition of data; analysis and interpretation of data; drafting of the manuscript. **Marcus Hentrich:** conception and design; acquisition of data; analysis and interpretation of data; drafting of the manuscript; supervision. **Franz Aschl:** analysis and interpretation of data; statistical analysis; critical revision of the manuscript. **Oksana Chernova:** analysis and interpretation of data; statistical analysis; critical revision of the manuscript. **Sebastian Beckstein:** acquisition of data; critical revision of the manuscript. **Peter Bojko:** acquisition of data; analysis and interpretation of data; critical revision of the manuscript. **Sebastian Schulz:** acquisition of data; analysis and interpretation of data; critical revision of the manuscript. **Julia Neitz:** acquisition of data; analysis and interpretation of data; critical revision of the manuscript. **Julian Hermans:** critical revision of the manuscript. **Yannic Volz:** critical revision of the manuscript. **Benedikt Ebner:** critical revision of the manuscript. **Patrick Keller:** critical revision of the manuscript. **Maria Apfelbeck:** critical revision of the manuscript. **Julian Marcon:** critical revision of the manuscript; supervision. **Philipp Weinhold:** critical revision of the manuscript. **Christian G. Stief:** critical revision of the manuscript; supervision; technical support. **Lennert Eismann:** critical revision of the manuscript. **Michael Chaloupka:** conception and design; analysis and interpretation of data; drafting of the manuscript.

## CONFLICT OF INTEREST STATEMENT

All authors declare that no conflicts exist.
